# Dr. Stokes, on M. Achard's Saccharine Beet-root

**Published:** 1800-01

**Authors:** Jonathan Stokes


### Dr. STOKES to Dr. BRADLEY.

DEAR SIR, *Chefterfield, Sept.* 22, 1799.

AS fome of the readers of the Medical and Phyfical Journal, in confequence of the account of Achard's experiments given at p. 188 of vol. i. may be induced to attempt to obtain fugar from the roots of beet, it may be well to inform them that M. Achard procured it from that kind which is generally known in this country by the name of *Mangold Wurzel*, or *Root of Scarcity*. M. Scherer, in his letter to Citoyen Van Mons, in the Annales de Chimie, No. 90, p. 299, calls it the "*Runkelrübe*, a plant which," he fays, "has been hitherto "ufed only as food for cattle," and his correfpondent, Van Mons, though he calls it a variety of the *Beta Cicla* of Linnæus, becaufe he does not find in the works of that author any variety with a white root arranged under *Beta vulgaris*, expreffly tells us, that the plant in queftion "is the *Betterave fur terre*, the "*Betterave champêtre*, *Racine d'abondance*, *Racine de difette* of "the French," and he defcribes "the leaves," as red, or "veined with red." "The Englifh," he adds, but on what authority he does not mention, "call it *Turlip*, which he thinks "led the news-writers, in fpeaking of Achard's difcovery, to fay "that he had obtained fugar from turnips." The *root of fcarcity*, which I cultivated fome years ago from feeds fent me by Dr. Lettfom, I referred to *Beta vulgaris*, from the flowers growing moftly four together, and from its general habit and colours, colours, "the ſpecific characters of the *Beta Cicla*" as Citoyen Van Mons obſerves, after Linnæus, "being, that the flowers "are in threes; and that of *Beta vulgaris*, that they are diſ-"poſed in a greater number together." A gentleman in this neighbourhood informs me, that he finds the roots the beſt winter food for milch cows, which it is natural to ſuppoſe they muſt be, if they afford ſo large a portion of ſugar. Do not neglect to inform the cow-keepers in the neighbourhood of London of this fact, as an acknowledgement for the readineſs with which they gave us all the information in their power on the ſubject of the Cow-pox.

I very much approve of your reſolution of not admitting anonymous ſtrictures on the obſervations of correſpondents who ſign their names; and I could wiſh you even to go farther, and reject all anonymous communications, unleſs containing narratives of unſucceſsful practice, or ſuggeſtions which a known individual might not dare to hazard, but which you might wiſh to make publicly known. In any diſcuſſions, too, between individuals, which might ſeem to border on the verge of controverſy, I could wiſh you to ſuppreſs every thing which might wear the leaſt appearance of perſonality. Another error you will have to guard againſt, is the ſpirit of diſſertation writing; and with reſpect to caſes, I hope you will refuſe to publiſh any whoſe termination is unknown, unleſs the author will engage to communicate, at ſome future period, the final reſult; and in cures of cancer, and ſome other chronic diſeaſes, it is your duty to take care that the reſult be communicated to the public after the lapſe of two or more years. The writers of caſes ſeldom publiſh new editions of their pamphlets, or if they do, they as ſeldom inform us of the preſent health of their patients,

I am, Dear Sir,

Yours, &c.

JONATHAN STOKES.

*Dr. B. aſſures his friend, Dr. Stokes, that his Communications, Hints, or Corrections, will always receive particular attention.*

